# Disrupted inhibin B synthesis due to Sox9 hyper-palmitoylation in Sertoli cells impairs spermatogenesis via paracrine effects

**DOI:** 10.1186/s40659-025-00661-y

**Published:** 2025-12-17

**Authors:** Yijian Xiang, Yao Xu, Jing Zhang, Miao Zhu, Zhaowanyue He, Ming Zang, Rujun Ma, Li Chen, Zhou Li, Tian Du, Liangyu Yao, Kuan Liang, Jiaming Shen, Shanmeizi Zhao, Jun Jing, Xie Ge, Bing Yao

**Affiliations:** 1 State Key Laboratory of Reproductive Medicine and Offspring Health, Nanjing Medical University, Nanjing, 211166 China; 2 Department of Reproductive Medicine, Jinling Hospital, Affiliated Hospital of Medical School, Nanjing University, Nanjing, 210002 China; 3 Department of Reproductive Medicine, Jinling Clinical Medical College, Nanjing University of Chinese Medicine, Nanjing, 210023 China; 4 Department of Cell Biology, Jinan University, Guangzhou, 510632 China; 5 The First School of Clinical Medicine, Southern Medical University, Guangzhou, 510515 China; 6 Department of Urology, Sichuan Provincial People’s Hospital East Sichuan Hospital, Dazhou First People’s Hospital, Dazhou, 635000 China; 7 Department of Reproductive Medicine, Jinling Hospital, Affiliated Hospital of Medical School, Southeast University, Nanjing, 210000 Jiangsu China; 8 Department of Urology, The First Affiliated Hospital of Nanjing Medical University, Nanjing, 210029 China

**Keywords:** Palmitic acid, Palmitoylation, SRY-box containing gene 9, Inhibin b, Spermatogenic microenvironment

## Abstract

**Background:**

Male subfertility is a global health concern, with spermatogenic dysfunction being a critical cause. Abnormally high level of palmitic acid (PA), a main component of dietary saturated fatty acid, has been reported to be implicated in the spermatogenic dysfunction, accompanied with a decrease of inhibin B (INHB). However, the mechanism underlying PA-induced downregulation of INHB, and the specific function of INHB in the spermatogenesis microenvironment, remain unclear. Since PA is the main substrate of palmitoylation, a common post-translational lipid modification, we investigated the role of palmitoylation in INHB synthetic defects and subsequent dyszoospermia induced by PA in this study.

**Methods:**

Mice were treated with PA for 30 days to establish a high PA model, and a palmitoylation inhibitor 2-bromopalmitate (2BP) was used for spermatogenesis rescuing. Concentrations and motilities of sperms in the cauda epididymides were analyzed, and pathological examinations were performed to assess spermatogenic function. Hormone levels were detected using ELISA. Primary mouse Sertoli cells and TM4 Sertoli cell line were used for in vitro exploration of mechanisms. Acyl biotin exchange assay was used to explore protein palmitoylation. Co-culture of TM4 and GC1 cells was used to explore the effects of Sertoli cell-secreted INHB on spermatogonia in a paracrine manner.

**Results:**

In this study, we found that excessive PA downregulates testicular INHB levels by suppressing expression of its βB subunit (InhβB) in Sertoli cells, with hyper-palmitoylation of the transcription factor SRY-box containing gene 9 (Sox9) serving as a key regulatory node in this process. We further identified the palmitoyl transferase ZDHHC16 as the primary enzyme responsible for PA-induced Sox9 hyper-palmitoylation. Furthermore, INHB was shown to promote spermatogonial proliferation and differentiation in a paracrine manner within the spermatogenic microenvironment, thereby mediating the modulation of spermatogenesis by palmitoylation in Sertoli cells.

**Conclusion:**

Overall, this study demonstrated that INHB synthesis can be suppressed by PA-induced hyper-palmitoylation of Sox9, and decreased secretion of INHB by Sertoli cells directly leads to spermatogenic dysfunction in the testis microenvironment. These findings highlight Sox9 palmitoylation as a candidate target for treatment of dyszoospermia accompanied with dyslipidemia, and underscore the critical role of INHB in regulating spermatogenesis within the testicular microenvironment.

**Supplementary Information:**

The online version contains supplementary material available at 10.1186/s40659-025-00661-y.

## Introduction

Infertility continues to pose a critical global health challenge, with male factors contributing to 30–50% of cases [1, 2]. Among the multifactorial etiologies of male infertility, spermatogenic dysfunction is a predominant pathogenic factor, and mounting evidence suggests that dysregulation of lipid metabolism is a key contributing factor [3]. Notably, elevated levels of saturated fatty acids, particularly the predominant saturated fatty acid palmitic acid (PA, C16:0), have been demonstrated to induce testicular lipotoxicity [4–6]. Since PA is widely present in processed foods and cooking oils, dietary exposure to PA is practically inescapable [7, 8]. Therefore, investigation of the mechanisms underlying PA-induced spermatogenic dysfunction and exploring possible therapeutic strategies is necessary.

In the spermatogenic microenvironment, Sertoli cells provide an indispensable homeostatic microenvironment through directly contacting with developing germ cells, providing paracrine factors and so on [9]. As a critical hormone produced by Sertoli cells, inhibin B (INHB) is a functional biomarker of Sertoli cell function and spermatogenic status [10]. Our previous studies demonstrated that excessive PA exposure induces a significant reduction in INHB levels, which is associated with dyszoospermia (i.e. abnormal semen parameters) [5, 6].

Elevated levels of PA, the main substrate for protein palmitoylation, is linked to dysregulated protein palmitoylation in multiple disease contexts. Protein palmitoylation is a reversible post-translational modification that involves thioester attachment of PA to cysteine residues, which is catalyzed by a family of zinc finger aspartate-histidine-histidine-cysteine (ZDHHC) protein acyltransferases [11]. Palmitoylation plays a critical role in regulating cellular processes such as protein trafficking, protein stability and signal transduction [12, 13], whereas aberrant palmitoylation patterns are causally linked to the pathogenesis of diverse diseases [14–16]. In our previously published study, hyper-palmitoylation has been demonstrated to mediate PA-induced blood-testis barrier destruction, indicating the involvement of palmitoylation in the regulation of Sertoli cell function [17]. However, the role of palmitoylation in the regulation of the synthesis and secretion of INHB in Sertoli cells remains to be explored.

Although it has been widely acknowledged that INHB exerts inhibitory effects on the secretion of follicle-stimulating hormone (FSH) and luteinizing hormone (LH) via the hypothalamic-pituitary-gonadal (HPG) axis, recent studies have also shed light on the localized functions of the hormones within the same family in specific microenvironments [18, 19]. The levels of INHB in testes have been reported to be associated with spermatogenic function [20], but the precise intratesticular mechanisms by which INHB modulates local spermatogenesis remain poorly understood. Furthermore, the potential impact of aberrant palmitoylation in Sertoli cells on spermatogenesis through modulation of INHB secretion is also elusive. Given that dyslipidemia has been implicated in male reproductive disorders, elucidating the role of INHB in the spermatogenic microenvironment and its regulation by palmitoylation pathways represents a critical research avenue.

In this study, we demonstrated that PA-induced protein hyper-palmitoylation in Sertoli cells could suppress the production of INHB by suppressing the transcription of its subunit *InhβB* gene. ZDHHC16-mediated hyper-palmitoylation of Sox9 was found to inhibit the transcription *InhβB* gene in Sertoli cells, which led to a decline in INHB production. Furthermore, our study unveils a critical role for INHB in the spermatogenic microenvironment, where it directly orchestrates spermatogonial proliferation and differentiation. Conversely, defects in INHB synthesis contribute to spermatogenic dysfunction under conditions of dysregulated palmitoylation. These findings indicate that aberrant Sox9 palmitoylation contributes to PA-induced disruption of INHB synthesis in Sertoli cells, which impairs spermatogenesis by a paracrine manner. These results provide novel insights into the mechanisms underlying spermatogenic dysfunction associated with metabolic disorders and suggest potential therapeutic targets for lipid-related male infertility.

## Materials and methods

### Animal model

Five-week-old male ICR mice obtained from the Experimental Animal Center of Nanjing Medical University were kept in a controlled environment with a temperature range of 21–26 °C for 12 h per light/dark cycle. In each experiment, mice were randomly assigned using the random number technique, forming three groups (*n* = 10 per group). The animal experiments were approved by the Animal Care and Use Committee of the Jingling Hospital (2023JLHGZRDWLS-000129), and were conducted according to the ARRIVE guideline for the Care and Use of Laboratory Animals. To establish the high PA mouse model, PA (Sigma-Aldrich, St. Louis, MO, USA) was administered intraperitoneally with a dose of 200 mg/kg/day for 30 days (conjugated with BSA, PA group). The selection of PA dosage is based on our previous published study, in which the dosage was cautiously selected and proved to be effective [17, 21, 22]. In the PA + 2BP group, mice were given a gavage of 2-bromopalmitate (2BP, Sigma-Aldrich) at a dose of 40 mg/kg every other day in combination with PA administration every day. The dosage of 2BP used in this study is a well-established and relatively specific concentration for inhibiting protein palmitoylation in vivo [17]. It has been demonstrated to effectively suppress the palmitoylation of various proteins without inducing significant off-target effects [23]. In the control group, mice were intraperitoneally injected with BSA and gavaged with saline.

## Assessing the concentrations and motilities of sperms

Sperms were carefully extracted from one of the cauda epididymidis of every mouse and diluted to 0.5 mL using HTF medium. After incubation at 37 °C for 5 min, sperm concentrations were assessed using a hemocytometer (Qiujing, Shanghai, China) and a light microscope (Olympus, Tokyo, Japan), with the data presented in millions per mL of suspension. Sperm motility was determined by counting the active spermatozoa within a designated area, which were presented as the percentage of motile sperm. To ensure blinded analysis, all images were assigned random codes by an independent researcher prior to analysis. Two observers, who remained blinded to the group allocation throughout the quantification process, then counted the sperm in ≥ 3 random fields per sample. The final data represent the average of both blinded counts.

## Histopathological observation

Mouse epididymides and testis were harvested and immediately fixed in 4% paraformaldehyde, embedded in paraffin and sectioned to 5-µm thickness. Afterwards, the sections were stained with hematoxylin and eosin (H&E) and observed using IX-73 microscope (Olympus Corporation, Tokyo, Japan) at appropriate magnification. The epithelial thickness of the seminiferous tubules in all mice were quantified. The Image J software from the National Institutes of Health in the United States was used to determine the thickness of tubules using images taken at 40× magnification.

## Immunohistochemistry (IHC)

Testicular tissue specimens were fixed in 4% paraformaldehyde, placed in 75% ethanol, dehydrated, and embedded in paraffin. Tissue sections with a thickness of 5 μm were exposed to anti-PCNA (ab92552, Abcam, 1:400) antibodies. Afterwards, the sections were cleansed and subsequently incubated with a secondary antibody, known as anti-rabbit immunoglobulin G. DAB staining was used to visualize protein expression. The IX73 fluorescence microscope (Olympus Corporation, Shinjuku, Tokyo, Japan) was used to capture and digitize the images.

## Immunofluorescence staining

Testicular tissue specimens were subjected to immunofluorescence staining as described [24]. Briefly, the testis sections were rehydrated with descending ethanol series and permeabilized using 0.1% Tween-20. After being blocked with 3% bovine serum albumin (BSA), the sections were incubated overnight at 4 °C with primary antibodies, including anti-LIN28 (ab46020, Abcam, 1:200) and anti-C-KIT (ab32363, Abcam, 1:500). Following secondary antibody incubation (50 min, dark), nuclei were counterstained with DAPI (C1005, Beyotime, 1:1000). LIN28^+^ or C-KIT^+^ cells were quantified across five random fields per section using an IX73 microscope, with ≥ 3 biological replicates per group.

Adherent cells were treated with 0.1% Triton X-100 and fixed for a duration of 20 min. Afterwards, cells were rinsed using PBS, followed by blocking with 5% goat serum in neutral PBS for 1 h at room temperature. Subsequently, cells were exposed to a rabbit polyclonal anti-Sox9 antibody (Ab5535, Millipore, 1:400) and incubated overnight at 4 °C. Additionally, the nuclei were stained with DAPI (1:1000). The images were captured under a laser scanning confocal microscope (LSM810, Zeiss, Germany; FV3000, Olympus, Japan). To ensure unbiased quantification, coded images acquired under standardized conditions were assessed by two independent, blinded observers. Using ImageJ, they counted positive and total cells in ≥ 3 random fields per sample, and their counts were averaged for the final data.

### Analysis of reproductive hormone levels in mouse sera, testes and cell culture medium supernatants

The blood samples from mice were centrifuged at room temperature for 10 min at 1,800×g, and then serum samples were taken. Testis homogenates and cell culture mediums were collected and centrifuged at 4 °C for 10 min at 4,000 rpm, and the supernatants were collected. The levels of triglyceride (TG) (Detection Range: 0.05–5 mg/mL), total cholesterol (TC) (Detection Range: 0.1–10 mmol/L), FSH (Detection Range: 2.5 to − 80 mIU/mL), LH (Detection Range: 0.25 mIU/mL − 8 mIU/mL), INHB (Detection Range: 1.5 to − 48 pg/mL) and testosterone (T) (Detection Range: 0.25 to − 8 ng/mL) in the samples were analyzed by ELISA kits (mlbio, Shanghai, China) according to the manufacturers’ instructions. All samples were run in duplicate. Assay precision, reflected in intra-assay coefficients of variation (CV), was rigorously monitored. The intra-assay CV (from sample duplicates) were consistently below 15%, thereby demonstrating excellent reproducibility. To preclude the potential impact of inter-assay CVs, all specimens were tested in a unified batch. Samples from all experimental groups were randomized across the plate(s) to prevent systematic bias.

## Isolation and culture of primary mouse Sertoli cells

Primary mouse Sertoli cells were isolated from 3-week-old ICR mice (Animal Center of Nanjing Medical University) through sequential enzymatic digestion. In brief, testicular tissues were sequentially digested with 0.25% trypsin (37 °C, 4–6 min) and 1 mg/ml collagenase I (37 °C, 6–8 min). The homogenate was filtered using 100-µm mesh to obtain single-cell suspension. After washing with PBS twice, the cells were cultured in DMEM/F12 supplemented with 10% FBS at 34 °C for 4 h. Then the unattached cells, which were mostly germ cells, were hypotonicly treated with 0.3×HBSS for 3 min, washed with PBS and discarded. The primary Sertoli cells were cultured for 48–72 h in vitro before experimentation.

## Culture of TM4 and GC-1 cell lines

TM4 cells (Procell Life Science & Technology Co. Ltd, Wuhan, China) were cultured in DMEM/F12 supplemented with 10% FBS. GC-1 cells (KE LEI Biological Technology Co. Ltd., Shanghai, China) were cultured in DMEM basic medium supplemented with 10% FBS. The cells were cultured under a 5% CO_2_ atmosphere at 37 °C.

### Assessment of cell viability and cell proliferation

Cell viability was analyzed using Cell Counting Kit-8 (C0037, Beyotime, Shanghai, China) according to the manufacturer’s instructions. Cell proliferation capacities were assessed using the BeyoClick^™^ EdU-488 Cell Proliferation Assay Kit (C0071S, Beyotime, Shanghai, China). Briefly, EdU working solutions in equal volumes were added to the cells, and incubation of the cells was continued for 2 h at 37 °C. Then the cells were fixed with 4% paraformaldehyde, and permeabilized with 0.2% PBST. Each well was added with Click reaction solution and incubated for 30 min at room temperature away from light. Images were obtained with an Olympus microscope under the light protection condition.

### Transfection of SiRNAs and plasmids

ZDHHC16-specific siRNAs (siZDHHC16), InhβB-specific siRNAs (siInhβB), as well as a non-specific siRNA (scrambled siRNA; negative control, NC) were designed and synthesized by GenePharma (Shanghai, China). Meanwhile, plasmids over-expressing Flag-tagged Sox9 and Flag-tagged InhβB were designed and synthesized by Uze Bio (Guangzhou, China).

For plasmid over-expression and siRNA knockdown, lipofectamine 3000 reagents (L3000015, Invitrogen, Thermo Fisher Scientific) was applied in the transfection process according to the guidelines provided by the manufacturer.

### Co-culture experiment

After transfection with siRNAs or plasmids, TM4 cells were cultured for 24 h. Meanwhile, GC-1 cells were placed in the bottom compartment of a transwell plate at a density of 1 × 10^5^ /well. The next day, TM4 cells were added to the top compartment of the transwell plate (0.4 μm). After incubation for 24 h, GC-1 cells were collected for protein extraction.

### RNA extraction and quantitative RT-PCR

The cells were subjected to total RNA extraction using the Total RNA Isolation Kit (082001, BEI-BEI Biotech, Zhengzhou, China), and cDNA was generated from total RNA by utilizing the PrimeScript RT reagent Kit with gDNA Eraser (RR047A, TaKaRa, Beijing, China). The cDNA was employed for quantitative PCR using SYBR Green Premix Pro Taq HS qPCR tracking kit (AG11733, Accurate Biotechnology Company, Changsha, China), and a Roche Light Cycler 96 Real-time PCR System (Roche Diagnostics, Switzerland) was used for fluorescence signal monitoring. The comparative ^ΔΔ^Ct method was utilized to calculate the relative expression with GAPDH as an internal control. The primer sequences used in this study are listed in Table 1.

**Table 1:** Primers used in RT–qPCR analyses

Gene	Forward primer	Reverse primer
*β-actin*	AGCCATGTACGTAGCCATCC	CTCTCAGCTGTGGTGGTGAA
*36B4*	GAAACTGCTGCCTTCACATCCG	GCTGGCACAGTGACCTCACACG
*InhβB*	CTTTGCAGAGACAGATGGCCT	GCCTGCACCACGAATAGGTT
*Inhα*	GGTGGGGATCCTGGAATAAG	GCACCTGTAGCTGGGAAAAG
*Zdhhc1*	ATGAACATCTGCAACAAACCCT	GCTCCATCCATTCCTTCGAGAG
*Zdhhc2*	TGTGCCTCATGGCTTATCATCT	TGGCATCTGTCACAATATCGGA
*Zdhhc4*	TGATTTGTGTTGTCCTGATCTGC	GGAGGCACTGCGGGATTAC
*Zdhhc6*	CATAGCCCTGGGTGTTATAGCA	CCTGAGACTTTTCCGGTTTCC
*Zdhhc9*	AAGGTGACACGGAAATGGGAG	CGACACTCGAAGGCAAAGAA
*Zdhhc11*	TGAGGTTACCGCGAGCAAAAA	CGAAGCTCATCAGGGTTGAC
*Zdhhc12*	CTCTCCTTCTTCGCGTTAGTG	CTGGCTTTTAGGCACAACAGC
*Zdhhc13*	TCGCAGTGCAGGAATCACAG	GGCAGCCCAGTGAAGAAGA
*Zdhhc14*	CACACTCTCAGACATTATGCCC	TGCATGGTACGGCTATGTGC
*Zdhhc16*	CAGCGAAGTCTGCTGTTGG	CAGCGGATCACATTGTCCAC
*Zdhhc19*	TGTGACACTTGTGAAGGAACC	AAAAACAGCAGCAGCGTTACA
*Zdhhc23*	ACTTGCGAAAGAATCACGGAT	ATGGGAAGGGAGGTCAGAACC
*Zdhhc24*	GCTTACTGTTACCAGTGCCAA	ACACAGCAGCCCAATAGGC

### Protein extraction and Western blot

Cells or tissues were rinsed with ice-cold 1×PBS and then immediately lysed in ice-cold RIPA (89901, Thermo Fisher Scientific) supplemented with 1 mM PMSF (ST506, Beyotime) and 1 mM protease inhibitor cocktail (complete Tablets EDTA-free, EASYpack, 04693132001, Roche). Protein concentrations were assessed using the Pierce BCA Protein Assay Kit (23225, Thermo Fisher Scientific). Equal amounts of protein samples were mixed with SDS-PAGE loading buffer, denatured by heating at 95 °C for 8 min, and then loaded onto PAGE gels for electrophoresis (ET15420Gel, ACE Biotechnology, Changzhou, China). Subsequently, the proteins were transferred to PVDF membrane (Merck Millipore, Billerica, MA, USA). The membranes were blocked for 1 h at room temperature in 5% BSA (4240GR250, BioFroxx GmbH), and incubated overnight at 4 °C with the primary antibodies, which were diluted in 5% BSA. The primary antibodies used in this study include: anti-PLZF (sc-28319, Santa Cruz, 1:200), anti-LIN28 (ab46020, Abcam, 1:1000), anti-C-KIT (ab32363, Abcam, 1:750), anti-STRA8 (ab49602, Abcam, 1:1000), anti-ZDHHC16 (bs-7677R, Bioss, 1:2000), anti-INHBB (TA350699S, ORIGENE, 1:2000), anti-Sox9 (Ab5535, Millipore, 1:2000), and anti-GAPDH (10494-1-AP, Proteintech, 1:50000). The membranes were washed three times with 1× Tris buffered saline containing 0.1% Tween-20 (TBST), each wash lasting 5 min. Subsequently, the membranes were incubated with the corresponding secondary horseradish peroxidase-conjugated antibody for 1 h at room temperature. After washing with 1× TBST for three times, the membranes were treated with SuperPico ECL Chemiluminescence Kit (E422-C1-P1, Vazyme, Nanjing, China) for visualization, and images were captured by Tanon-5200 Chemiluminescent Imaging System (Tanon Science and Technology, Co., Ltd., Shanghai, China). The target band intensities were quantified using ImageJ software (National Institutes of Health, MD, USA) to calculate the relative protein expression levels. To ensure objective analysis, the quantification process was carried out in a blinded fashion. The original blot images were renamed and randomized by one investigator before being provided to another investigator who was blinded to the sample identities. The band intensity of the target protein was normalized to the corresponding loading control GAPDH.

### Acyl biotin exchange (ABE) assay

To evaluate the palmitoylation of endogenously expressed proteins, a method using HPDP-Biotin was executed as described in published article [17, 25]. The protein was irreversibly blocked with unmodified cysteine thiol groups with NEM (50 mM, 4 °C in the dark, overnight). After precipitation with methanol/chloroform, the protein samples were diluted in 200 µL resuspension buffer (4% SDS, 50 mM Tris and 5 mM EDTA, pH 7.4), and each sample was split into two equal parts (100 µL per part): plus-HAM (+ HAM) sample and minus-HAM (− HAM) sample. The + HAM sample was mixed with 800 µL of 1 M HAM, 1 mM EDTA, protease inhibitors and 100 µL of 4 mM HPDP-biotin. The negative control group (− HAM sample) was treated identically, but HAM was replaced by 50 mM Tris (pH 7.4). Samples were incubated while rotating at room temperature (RT) in the dark for 2 h, and then precipitated again and dissolved in 100 µL of resuspension buffer. Afterwards, 900 µL PBS containing 0.2% Triton X-100 and 15 µL streptavidin-coated beads were added with shaking overnight at 4 °C. The beads were washed five times with PBS. Finally, the beads were mixed with 2× SDS loading buffer and boiled at 95 °C for 5 min. Then, the samples were analyzed by Western blot.

To evaluate the palmitoylation of exogenously expressed Flag-tagged Sox9, the ABE assay was used after immunoprecipitation using anti-Flag beads, as previously described [17]. Briefly, HEK-293T cells transiently transfected with the plasmid expressing Flag-tagged Sox9 were harvested and washed with cold PBS. Then the cells were suspended in lysis buffer (LB, 50 mM Tris-HCl, 150 mM NaCl, 1 mM MgCl_2_, 1% NP-40, 10% glycerol, pH = 7.5) containing 50 mM N-ethylmaleimide (SLCK9172, MilliporeSigma), 1 mM PMSF and 1 mM protease inhibitor cocktail. After lysing in LB for 1 h at 4 °C, the cells were centrifuged at 4 °C, 13,000×g to remove insoluble materials. The supernatants were incubated with anti-Flag beads (B26101, Selleck, USA) at 4 °C overnight. Then the beads were washed five times, and each sample was divided into two parts, with one part being incubated with LB (pH = 7.2) containing 1 M hydroxylamine (HAM, 467804, MilliporeSigma, ) at room temperature for 1 h (+ HAM), while the other part omitting the HAM incubation step (-HAM). After being washed four times with LB (pH = 7.2) and twice with LB (pH = 6.2), the beads were treated with LB (pH = 6.2) containing 50 mM biotin-BMCC (C100222-0050, Sangon Biotech, Shanghai, China) at 4 °C for 1 h. Then all samples were gently washed twice in LB (pH = 6.2) and three times in LB (pH = 7.5). The immunoprecipitate samples were analyzed by immunoblot using HRP-streptavidin (RABHRP3, sigma, 1:10000).

### Prediction of S-palmitoylation sites and nuclear localization signals

The prediction of S-palmitoylation sites was performed by CSS-Palm 4.0 (http://csspalm.biocuckoo.org/) [26], a deep learning-based graphic presentation system for the prediction of S-palmitoylation sites in proteins.

We predicted the zDHHCs nuclear localization signals (NLS) specific to the importin pathway by cNLS Mapper (https://nls-mapper.iab.keio.ac.jp/cgi-bin/NLS_Mapper_form.cgi) [27], based on the amino acid sequence of each protein.

### Statistical analysis

All data are presented as mean ± standard deviation (SD) from at least three independent experiments. Statistical analysis was performed using GraphPad Prism 9.0. The normality of data distribution for each experimental group was first assessed using the Shapiro-Wilk test, and all datasets passed the normality assumption (*p* > 0.05). For comparisons between two groups, an unpaired two-tailed Student’s *t*-test was used. For comparisons among more than two groups, one-way analysis of variance (ANOVA) was employed. For comparisons among more than two groups not in accordance with the normal distribution, ann-Whitney U test (two groups) or Kruskal-Wallis test with Dunn-Bonferroni correction was employed (*n* ≥ 3 groups). Effect sizes for significant findings were calculated as Cohen’s d for *t*-tests and 1-β for ANOVA. Exact p-values are reported throughout the results section, and the threshold for statistical significance was set at *p* < 0.05. The raw data for all graphs and analyses have been provided as a EXCEL 2-Statistic Data file. Graphical Figure was created with BioRender.com and CorelDRAW X7 Version 17.0.0.491.

## Results

### PA-induced hyper-palmitoylation impaired spermatogenesis in mice

**Fig. 1:**
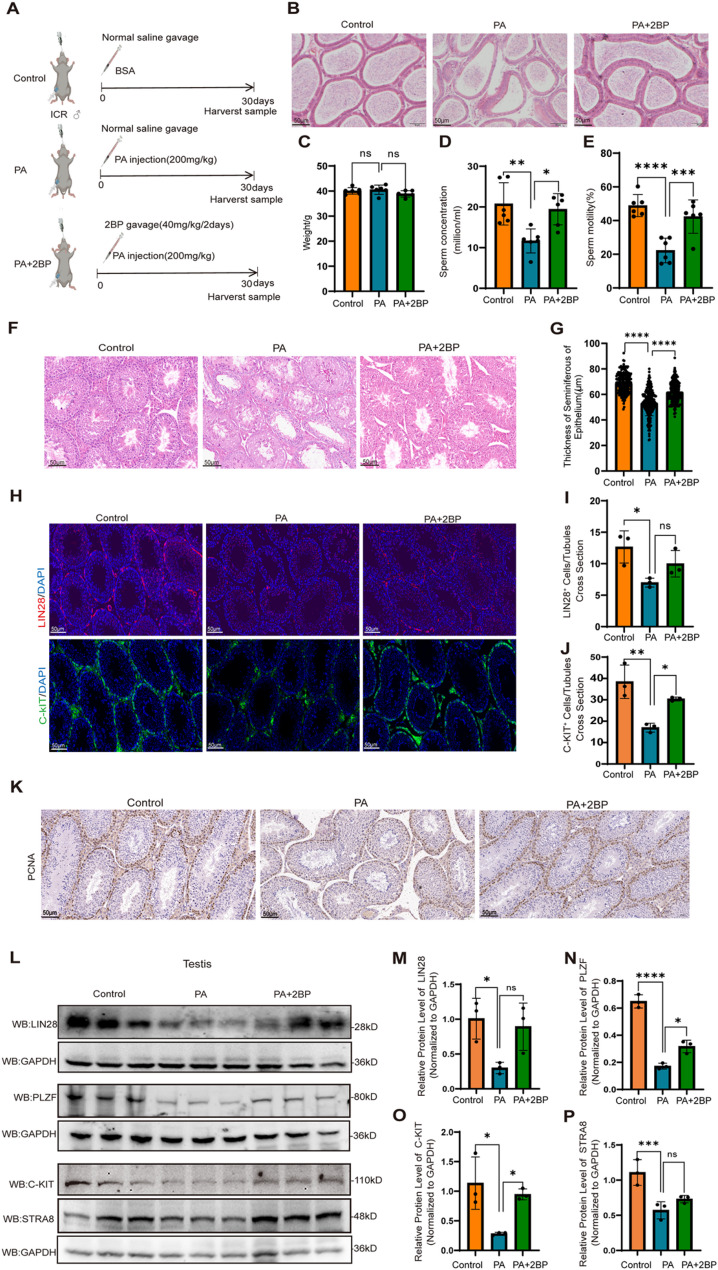
PA disrupted spermatogonial proliferation and differentiation and spermatogenesis. **A** Animal model establishment and treatment protocols. **B** Representative H&E-stained sections of epididymal tails harvested from mice in the control, PA, and 2BP-treated groups (*n* = 3). Scale bar: 50 μm. **C** The weight of mice (*n* = 6). **D** and **E** Analysis of sperm concentrations (**D**) and motilities (**E**) (*n* = 6). **F** and **G** Representative **H** and **E** staining images and transmission electron microscopy images of testicular cross-sections from each group (**F**) (*n* = 4 per group; scale bar, 50 μm). Comparison between the model mice and control mice involves assessing the quantity of seminiferous tubules exhibiting empty, partial or full spermatogenesis (**G**). **H** Immunofluorescence staining of LIN28 (red) and C-KIT (green) (scale bar 50 μm) (*n* = 3). **I** and **J** Counts of LIN28^+^ (**I**) and STRA8^+^ (**J**) cells per seminiferous tubule cross-section in testicular biopsies (*n* = 3). Comparison with control: *p < 0.05; **p < 0.01; ***p < 0.001. **K** Representative IHC images of PCNA in testes from control, PA and PA + 2-BP mice (*n* = 3). **L**–**P** Western blot of C-KIT, STRA8, PLZF, and LIN28, with GAPDH used as the internal control. Representative images of Western blot results were presented (**L**), and relative expression levels of these proteins were assessed (**M**–**P**) (*n* = 3). Data are shown as mean ± SEM from at least three independent experiments. Comparison with control: *p < 0.05; **p < 0.01; ***p < 0.001

To explore the impact of PA-induced hyper-palmitoylation on spermatogenesis, an in vivo experiment was conducted. Five-week-old mice were intraperitoneally injected with PA and treated with or without the palmitoylation inhibitor 2BP by gavage (Fig. 1A). After 30 days of treatment, epididymides were collected from mice in each group. H&E staining of paraffin-embedded caudal epididymal sections showed a significant reduction of sperm counts in epididymal tubules in the PA-treated group, which was partially restored by 2BP treatment (Fig. 1B). Meanwhile, the body weight of the mice was measured, and it was found that there was no significant change (Fig. 1C). We also analyzed the concentrations and motilities of sperm in the caudal epididymides. Compared to controls, sperm concentrations and motilities were both significantly reduced in the PA-treated group, but were restored to near-normal levels in the 2BP + PA group (Fig. 1D, E). Meanwhile, we observed the seminiferous tubules in the testes by H&E staining (Fig. 1F). The epithelial thickness of the seminiferous tubules were assessed. The result indicated that the seminiferous tubule thickness was significantly reduced in the high PA group compared to the control, which were restored in the PA + 2BP group (*p* < 0.05) (Fig. 1G).

Given spermatogonia are the starting point for spermatogenesis, we further examined whether PA-induced hyper-palmitoylation affects the proliferation and differentiation capabilities of spermatogonia. Because spermatogonia can be classified into undifferentiated spermatogonia and differentiated spermatogonia, we conducted immunofluorescence staining on testicular sections from model mice to further investigate the specific spermatogonia type affected by PA, and the undifferentiated spermatogonia-specific biomarker protein lin-28 homolog (LIN28) and differentiated spermatogonia-specific biomarker KIT proto-oncogene receptor tyrosine kinase (C-KIT) were stained (Fig. 1H). The results showed that PA reduced both the numbers of undifferentiated spermatogonia (LIN28^+^) and differentiated spermatogonia (C-KIT^+^) in the seminiferous tubules, and 2BP had a significant rescuing effect (Fig. 1I, J).

Considering that spermatogonia are the only spermatogenic cells undergoing mitotic proliferation, we assessed the effect of PA treatment on spermatogonial proliferation. Immunohistochemistry staining for the proliferation marker proliferating cell nuclear antigen (PCNA) showed that testes from mice in the PA group had fewer PCNA-positive spermatogonia than those from mice in the control group, which were restored in the PA + 2BP group (Fig. 1K).

Western blot analysis was conducted to validate the protein expression of the biomarkers of undifferentiated spermatogonia (PLZF, LIN28) and differentiated spermatogonia (C-KIT, STRA8). The results showed that the expression of both undifferentiated and differentiated spermatogonia biomarkers was decreased in the testes of PA-treated mice compared to controls, which was restored by 2BP treatment (Fig. 1L-P). Collectively, these findings suggest that PA-induced protein hyper-palmitoylation primarily impairs spermatogenesis by inhibiting spermatogonial proliferation and differentiation.

### PA-induced hyper-palmitoylation decreased INHB levels in the testes

**Fig. 2:**
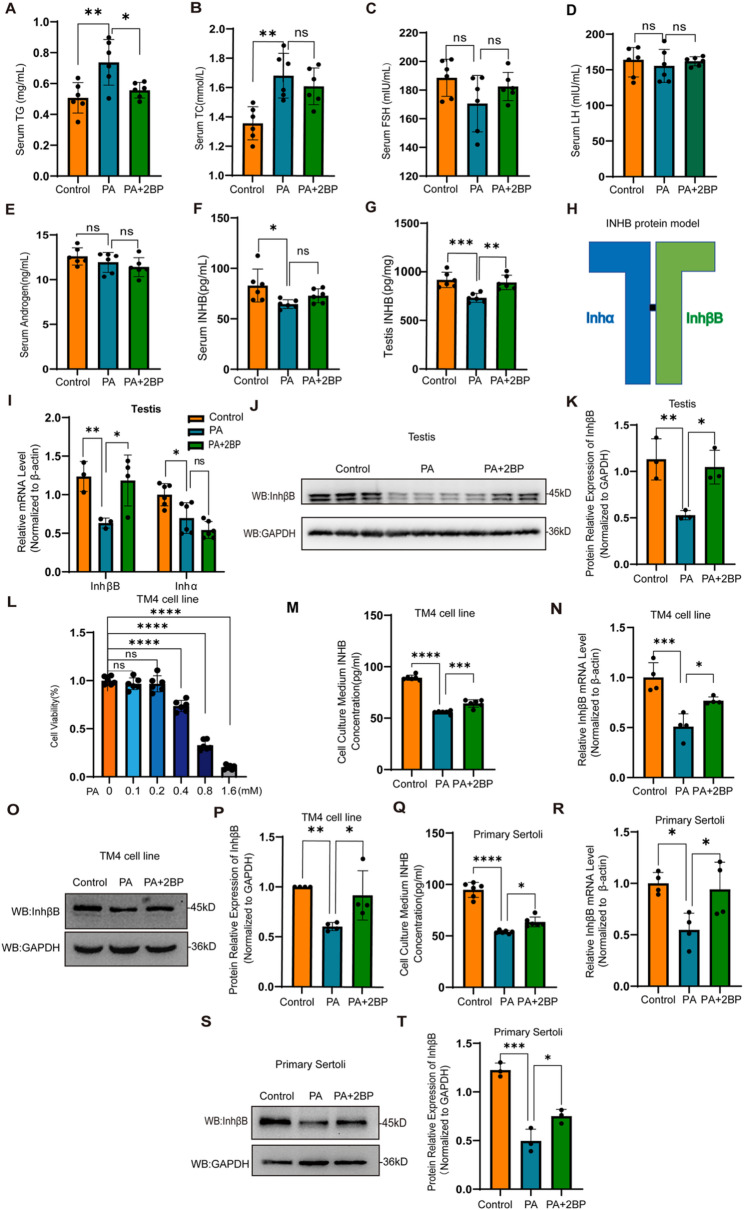
PA-induced protein hyper-palmitoylation reduced the synthesis of INHB. **A**–**F** Serum TG (**A**), serum TC (**B**), serum FSH (**C**), serum LH (**D**), serum T (**E**) and serum INHB (**F**) levels in mice were measured by ELISA assay (*n* = 6). **G** The levels of INHB in mouse testis were analyzed by ELISA assay in each group (*n* = 6). **H** Structure diagram of INHB (heterodimer of Inhα and InhβB subunits). **I** RT-qPCR detection of testicular expression level of Inhα and InhβB mRNA (*n* = 3). **J** The protein level of InhβB in testes was detected by Western blot (*n* = 3). **K** Relative quantification of the protein expression level of InhβB (*n* = 3). **L** CCK-8 detection of the cell viability of TM4 cells treated with PA in gradient concentration (0.1 to 1.6 mM). **M** Concentrations of INHB in culture medium supernatants of TM4 cells were analyzed by ELISA. TM4 cells were treated with PA (0.4 mM) and PA + 2BP (10 µM) for 24 h (*n* = 6). **N** After TM4 cells were treated with PA (0.4 mM) and PA + 2BP (10 µM) for 24 h, the expression of InhβB mRNA was assessed using RT-qPCR for mRNA (*n* = 4). **O** The protein level of InhβB in TM4 cells was assessed by Western blot. **P** Relative quantification of the protein expression of InhβB (*n* = 4). **Q** Concentrations of INHB in culture medium supernatants of primary Sertoli cells were analyzed by ELISA assay, treated with PA (0.4 mM) and PA + 2BP (10 µM) (*n* = 6). **R** After primary Sertoli cells were treated with PA (0.4 mM) and PA + 2BP (10 µM) for 24 h, the expression of InhβB mRNA was assessed with RT-qPCR (*n* = 4). **S** Protein levels of InhβB in primary Sertoli cells were detected by Western blot. **T** Relative quantification of protein expression of InhβB was determined (*n* = 3)

To explore the impact of PA-induced hyper-palmitoylation on the lipid metabolism of mice, we detected the serum lipid levels. The results showed that the levels of triglyceride (TG) and total cholesterol (TC) were increased in the PA group compared with controls(Fig. 2A, B), suggesting that administration of PA successfully induced hyperlipidemia, and the elevated serum lipid levels serve as an indirect indicator of successful model establishment. Moreover, FSH and LH are key regulators of the HPG axis and serve as established biomarkers in the clinical diagnosis of dyszoospermia. In this study, we measured serum FSH and LH levels in a mouse model with elevated PA. As shown in Fig. 2C and D, neither FSH nor LH level was significantly altered under high-PA conditions. These results lead us to explore the alterations in the testicular microenvironment induced by high PA treatment.

In the testicular spermatogenic microenvironment, Leydig cells and Sertoli cells are two key somatic cell types that maintain normal spermatogenesis by secreting active substances, providing structural support, and regulating signals. Therefore, we measured serum levels of the reproductive hormones T, a critical hormone secreted by Leydig cells, and INHB, a critical hormone secreted by Sertoli cells. The experimental results did not show significant differences of serum T levels among the three groups of mice (Fig. 2E), indicating that Leydig cells do not likely play a key role in PA-induced blockage of spermatogonia proliferation and differentiation. Serum INHB levels were significantly reduced in the PA group compared to controls (Fig. 2F), suggesting that Sertoli cells may contribute to PA-induced spermatogenic disorders. However, 2BP treatment did not fully restore the PA-induced decline in serum INHB, but there is a certain possibility that the salvage effect of 2BP was obscured by dilution in the bloodstream. Therefore, we further examined whether PA affected local INHB levels in the testes. ELISA analysis revealed that 2BP treatment restored the PA-induced reduction of INHB levels in mouse testes (Fig. 2G).

INHB is consisted of two subunits, Inhα and InhβB (Fig. 2H), and InhβB was previously reported to be the limiting factor in the production of bioactive inhibin in Sertoli cells [28, 29]. We analyzed the mRNA levels of InhβB and *Inhα* in the testes using RT-qPCR, and found that PA treatment significantly decreased the transcript levels of both subunits, while treatment of a palmitoylation inhibitor 2BP only restored the PA-induced reduction of InhβB mRNA (Fig. 2I). Western blot analysis further confirmed that InhβB mRNA expression was reduced by PA on the protein levels, and 2BP showed an obvious rescuing effect (Fig. 2J, K). These results indicate that PA-induced protein hyper-palmitoylation down-regulated local INHB levels in the testis by suppressing the expression of InhβB mRNA.

To establish an effective dosing regimen, TM4 cells were treated with PA, and cell viability was measured using the CCK-8 assay. We conducted a pilot screen across a broad concentration range of PA from 0.1 mM to 1.6 mM. This simulates the concentration of PA in the human circulatory system and in the mouse serum [5, 30–32]. Based on the results of this preliminary screen, we have ascertained that the effective concentration range of PA, which triggers a significant reduction in cell viability, spans from 0.4 mM to 1.6 mM (Fig. 2L). As 0.4 mM of PA induced significant but not dramatic cell death, we selected 0.4 mM for all subsequent in vitro studies.

To elucidate whether protein palmitoylation affects the secretion of INHB by Sertoli cells in vitro, we measured the concentrations of INHB in the culture supernatants of TM4 Sertoli cells and primary Sertoli cells using an ELISA assay. The results showed that PA significantly decreased the concentrations of INHB in both cell types. Notably, treatment with 2BP effectively reversed the PA-induced reduction in INHB levels (Fig. 2M, Q). We furtherly examined the effects of palmitoylation on the expression levels of InhβB mRNA in TM4 cells and primary Sertoli cells, and found that PA treatment significantly decreased the expression levels of InhβB mRNA compared to controls, while 2BP restored InhβB mRNA levels (Fig. 2N, R), which is in accordance with our in vivo experiment results. Western blot analysis further showed that PA reduced InhβB protein expression in both TM4 and primary Sertoli cells, which was rescued by 2BP treatment (Fig. 2O, P, S, T). Collectively, these results indicate that protein palmitoylation modulates INHB synthesis in Sertoli cells by affecting the transcription of *InhβB* gene.

### PA induces hyper-palmitoylation and attenuates nuclear localization of Sox9, a transcription factor of InhβB

**Fig. 3:**
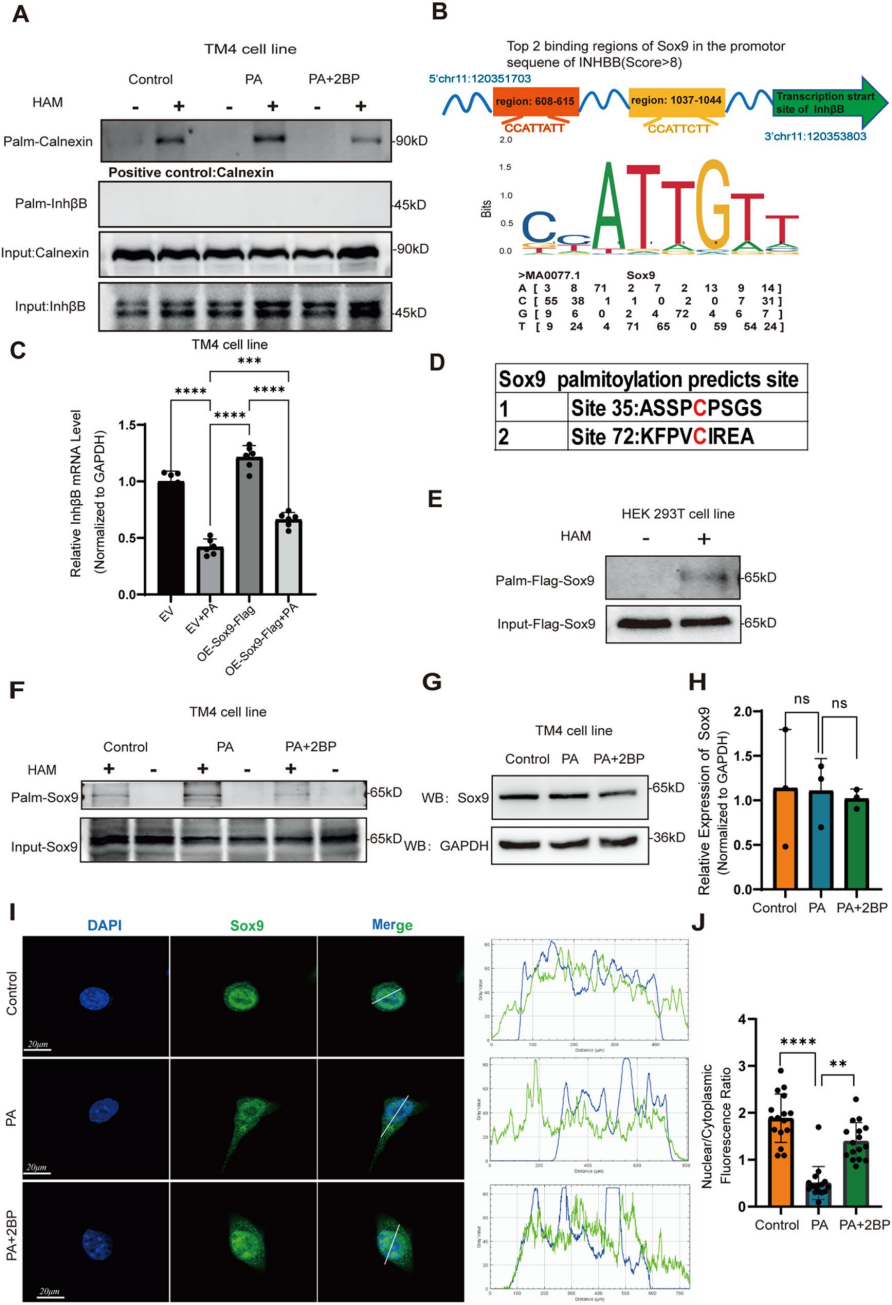
PA induces hyper-palmitoylation and attenuates nuclear localization of Sox9, a transcription factor of InhβB. **A** The palmitoylation levels of InhβB were detected by ABE assay, after TM4 cells were treated with PA (0.4 mM) and PA + 2BP (10 µM) for 24 h. **B** Predicted binding regions of Sox9 in the promoter of *InhβB* gene. **C** The expression of InhβB mRNA was detected by RT-qPCR after overexpressing Flag-Sox9 and treatment with PA in TM4 cells (*n* = 6). **D** Predicted palmitoylation sites in Sox9. **E** Palmitoylation of Flag-Sox9 was detected by ABE assay. **F** The palmitoylation levels of Sox9 in TM4 cells treated with PA (0.4 mM) and 2BP (10 µM) were determined by ABE assay. **G** The protein expression of Sox9 was determined by Western blot. **H** Relative quantification of the protein expression level of Sox9 (*n* = 3). **I** The changes in the nuclear localization of Sox9 was detected by immunofluorescence staining. **J** The fluorescence ratio of nuclear/cytoplasmic Sox9 in TM4 cells treated with PA (0.4 mM) and 2BP (10 µM) (*n* = 16)

As we demonstrated that protein palmitoylation is involved in PA-induced suppression of INHB synthesis in Sertoli cells through downregulating the expression of InhβB mRNA, we further investigated the specific palmitoylated proteins involved in this process. Firstly, we examined whether the critical subunit of INHB, InhβB, could be directly modified by palmitoylation. We assessed the palmitoylation status of InhβB using the ABE assay, with palmitoylated Calnexin serving as a positive control. The results indicated that InhβB was not palmitoylated (Fig. 3A), suggesting that there may be other palmitoylated proteins involved in this process.

According to our results, PA-induced downregulation of INHB levels is mediated by the disruption of transcription of *InhβB* gene. Thus, we hypothesized that PA-induced hyper-palmitoylation may affect the expression or activity of some transcription factor of *InhβB* gene. Sox9 is an important marker of Sertoli cells, which has been reported to play a critical role in the regulation of spermatogenesis by Sertoli cells [33]. Previous studies have identified Sox9 as a transcription factor promoting the transcription of *InhβB* gene [34]. Using the transcription factor prediction website JASPAR. We found that Sox9 may bind to the promoter region of *InhβB* gene (Fig. 3B). We overexpressed Sox9 in TM4 cells and demonstrated that the mRNA level of InhβB, which was downregulated by PA, could be rescued (Fig. 3C). These results validated that Sox9 acts as a transcription factor to promote InhβB mRNA expression.

Therefore, we predicted palmitoylation sites in Sox9 using the palmitoylation site prediction website CSS-Palm (http://csspalm.biocuckoo.org/), and two palmitoylation sites, C35 and C72, were identified (Fig. 3D). We constructed Flag-tagged Sox9 overexpressing plasmid, transfected it into HEK-293T cells, and detected the palmitoylation level of Flag-Sox9 by ABE assay. The experimental results showed that Sox9 could be palmitoylated (Fig. 3E). In addition, we confirmed that the palmitoylation of Sox9 was promoted by PA and inhibited by 2BP (Fig. 3F).

Previous studies have demonstrated that palmitoylation can modulate protein expression and subcellular location [35]. We firstly examined the effects of PA and 2BP on Sox9 protein expression in TM4 cells using Western blot, and found that PA and 2BP treatment did not significantly change Sox9 expression on the protein level (Fig. 3G, H). Then we investigated whether aberrant palmitoylation of Sox9 could affect its nuclear localization. Sox9 is typically localized to the nucleus, where it activates the transcription of downstream genes, so we focused on the change of Sox9 nuclear localization. Cellular immunofluorescence experiments showed that PA treatment reduced the nuclear translocation of Sox9, while 2BP showed a rescuing effect (Fig. 3I). These findings imply that PA-induced hyper-palmitoylation attenuates nuclear localization of Sox9, which may thereby affect the transcription of *InhβB* gene.

#### ZDHHC16 is speculated as a key palmitoyltransferase regulating Sox9 palmitoylation

**Fig. 4:**
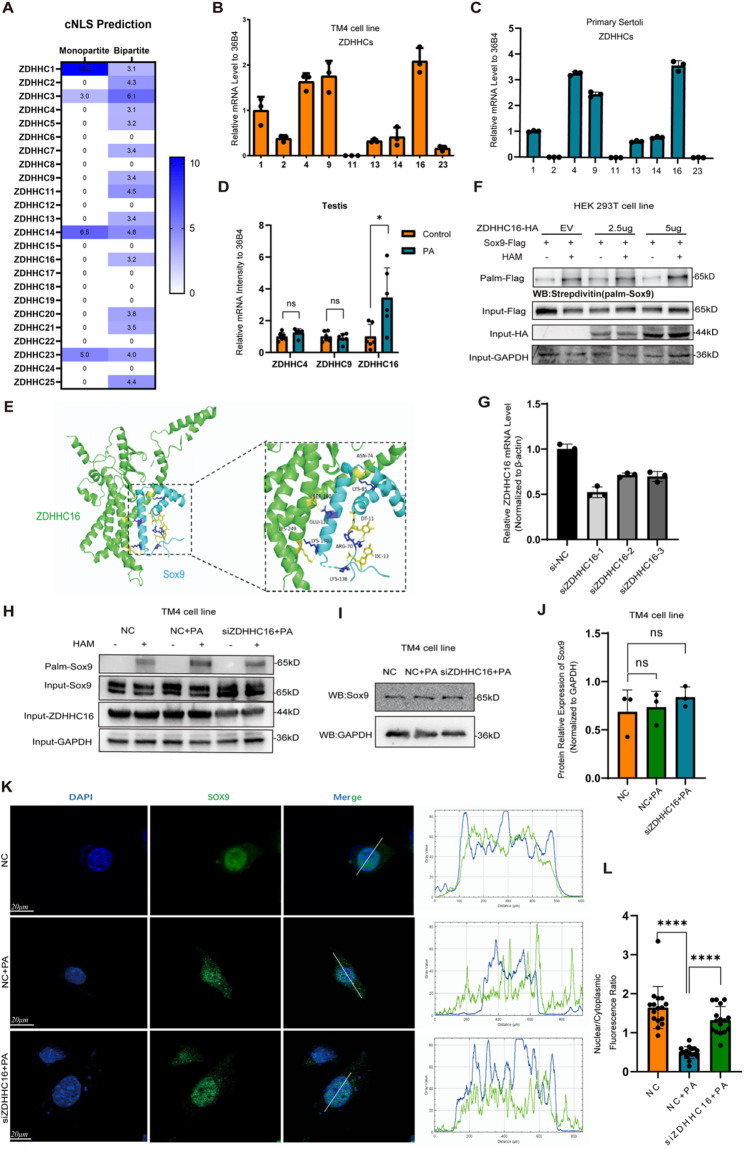
ZDHHC16 is a key palmitoyltransferase regulating Sox9 palmitoylation. **A** Prediction of the nuclear location signal of ZDHHCs. **B** Relative expression of palmitoyltransferases localized in the endoplasmic reticulum in TM4 cells by RT-qPCR (*n* = 6). **C** Relative expression of palmitoyltransferases localized in the endoplasmic reticulum in primary Sertoli cells by RT-qPCR (*n* = 3). **D** The mRNA expression of ZDHHC4, ZDHHC9, and ZDHHC16 in the mouse testes was detected by RT-qPCR (*n* = 3). **E** Molecular docking model of ZDHHC16 and Sox9 (predicted by AlphaFold 3 software). **F** The palmitoylation levels of Flag-Sox9 were determined by exogenous ABE assay. **G** The knockdown efficiency of ZHHC16 by siRNAs was verified by RT-qPCR (*n* = 3). **H** The palmitoylation levels of Sox9 were detected by ABE assay. **I** The protein expression levels of Sox9 were detected by Western blot. **J** Relative quantification of the protein expression levels of Sox9 (*n* = 3). **K** Detection of the changes of nuclear localization of Sox9 by cellular immunofluorescence. **L** The fluorescence ratio of nuclear/cytoplasmic Sox9 in TM4 cells (*n* = 16)

Protein palmitoylation is catalyzed by ZDHHC protein acyltransferases, so we further explored the specific acyltransferase that promotes the palmitoylation of Sox9 in the presence of excess PA. Our previous findings indicated that PA is internalized by Sertoli cells and initially localized to the endoplasmic reticulum (ER) [17]. Given this subcellular localization, we hypothesized that PA may interact with ZDHHC acyltransferases primarily located in the ER. On the other hand, Sox9 is predominantly localized in the nucleus. Therefore, we hypothesize that palmitoylation of Sox9 may be catalyzed by a ZDHHC acyltransferase capable of shuttling between the ER and the nucleus. Among the ZDHHCs, 13 have been shown to localize to the ER, including ZDHHC1, 2, 4, 6, 9, 11, 12, 13, 14, 16, 19, 23, and 24 [17]. We further predicted the nucleus localization signals (NLS) of these ZDHHCs to assess their potential to translocate into the nucleus using the NLS database. Based on these predictions, ZDHHC1, 2, 4, 9, 11, 13, 14, 16, and 23 were selected (Fig. 4A).

We then screened the expression levels of these ZDHHCs mRNA in TM4 and primary Sertoli cells using RT-qPCR. The results revealed that ZDHHC16 mRNA was most highly expressed, with relatively high expression levels also observed for ZDHHC4 and ZDHHC9 (Fig. 4B, C). Moreover, the mRNA expression of ZDHHC16 was significantly upregulated in the PA-treated group compared to controls (Fig. 4D). Based on these findings, we presumed that ZDHHC16 may be involved in PA-induced hyper-palmitoylation.

We used the small-molecule docking prediction model AutoDock (https://www.cgl.ucsf.edu/chimera/data/downloads/1.7/docs/ContributedSoftware/vina/vina.html) to predict the potential interactions between ZDHHC16 and Sox9 [36]. The results indicated a strong interaction between ZDHHC16 and Sox9 (Fig. 4E). After transfecting HEK-293T cells with a HA-tagged ZDHHC16 overexpressing plasmid at varying concentrations (0, 2.5, and 5 µg), we found that the palmitoylation level of Sox9 was increased by ZDHHC16 overexpression in a dose-dependent manner (Fig. 4F).

To further validate the regulation of Sox9 palmitoylation by ZDHHC16, we knocked down ZDHHC*16* using siRNA. Three duplexes of ZDHHC16 siRNA and a scrambled siRNA were designed and synthesized, and among the three duplexes, siZdhhc16-1 exhibited the most effective silencing effect (Fig. 4G). Thus, siZDHHC16-1 was used in subsequent experiments. The results revealed that ZDHHC16 knockdown reduced the hyper-palmitoylation of Sox9 induced by PA (Fig. 4H). Hence, we hypothesized that ZDHHC16 may serves as the pivotal enzyme regulating the palmitoylation of Sox9.

Previously, we found that PA-induced hyper-palmitoylation impairs Sox9 nuclear localization, but shows no significant effect on Sox9 expression. By knocking down ZDHHC16 in TM4 cells using siRNA, we confirmed the effect of reduced Sox9 palmitoylation on its expression and subcellular localization. The results presented that ZDHHC16 knockdown did not rescue the PA-induced decrease of Sox9 expression (Fig. 4I, J), but rescued the nuclear translocation of Sox9, which was attenuated by PA (Fig. 4K). These results suggest that ZDHHC16 can be considered a key enzyme catalyzing the palmitoylation of Sox9 in the presence of excess PA, which affects the nuclear localization of Sox9.

**ZDHHC16 knockdown ameliorated PA-induced INHB synthesis defects by restoring the transcription of** ***InhβB*** **gene**.

**Fig. 5:**
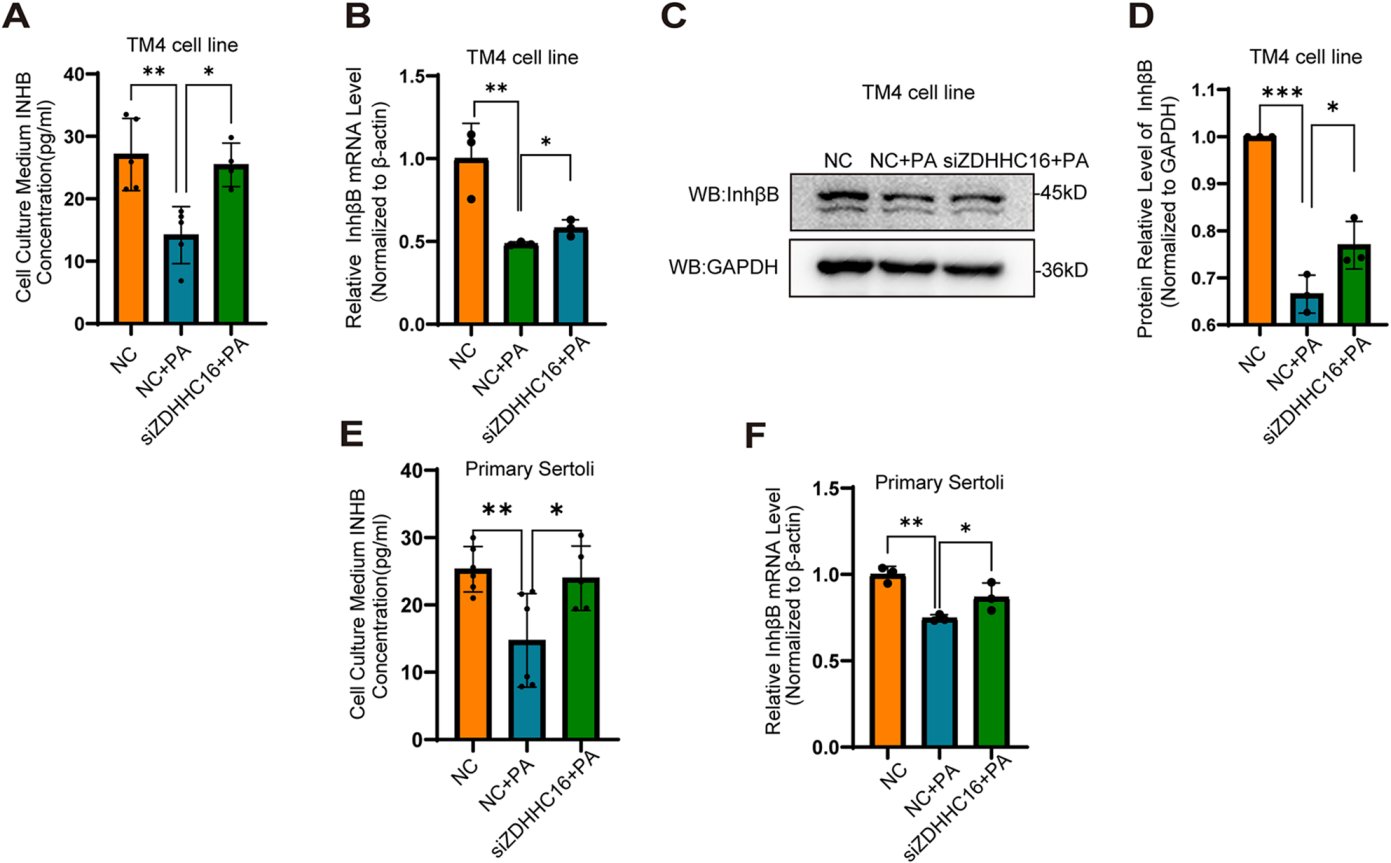
ZDHHC16 knockdown ameliorated PA-induced INHB synthesis defects by restoring the transcription of *InhβB* gene. **A**, **E** The INHB levels in the culture medium supernatants were determined by ELISA. TM4 cells and primary Sertoli cells were transfected with ZDHHC16-specific siRNA and then treated with PA (0.4 mM) for 24 h (*n* = 6). **B** The mRNA levels of InhβB were determined by RT-qPCR. TM4 cells were transfected with ZDHHC16-specific siRNA for 24 h (*n* = 3). **C** The protein expression levels of InhβB were detected by Western blot. TM4 cells were transfected with ZDHHC16-specific siRNA for 24 h. **D** The relative protein quantification of InhβB was determined by grayscale analysis (*n* = 3); **F** The mRNA levels of InhβB were determined by RT-qPCR. Primary mouse Sertoli cells were transfected with ZDHHC16-specific siRNA (*n* = 3)

To determine whether ZDHHC16, which catalyzes Sox9 palmitoylation, could affect INHB levels in Sertoli cells, we knocked down ZDHHC16. Our results showed that in both TM4 and primary Sertoli cells, ZDHHC16 knockdown significantly restored the INHB levels in the cell culture medium supernatant, which were decreased by PA treatment (Fig. 5A, E). ZDHHC16 knockdown also reversed the PA-induced downregulation of InhβB mRNA (Fig. 5B, F), and the protein expression level of InhβB also showed a similar variation tendency (Fig. 5C, D). Collectively, these findings demonstrate that ZDHHC16 regulates INHB levels by modulating the transcription of *InhβB.*

### Disrupted INHB synthesis induced by Sox9 hyper-palmitoylation affects spermatogonial proliferation and differentiation

**Fig. 6:**
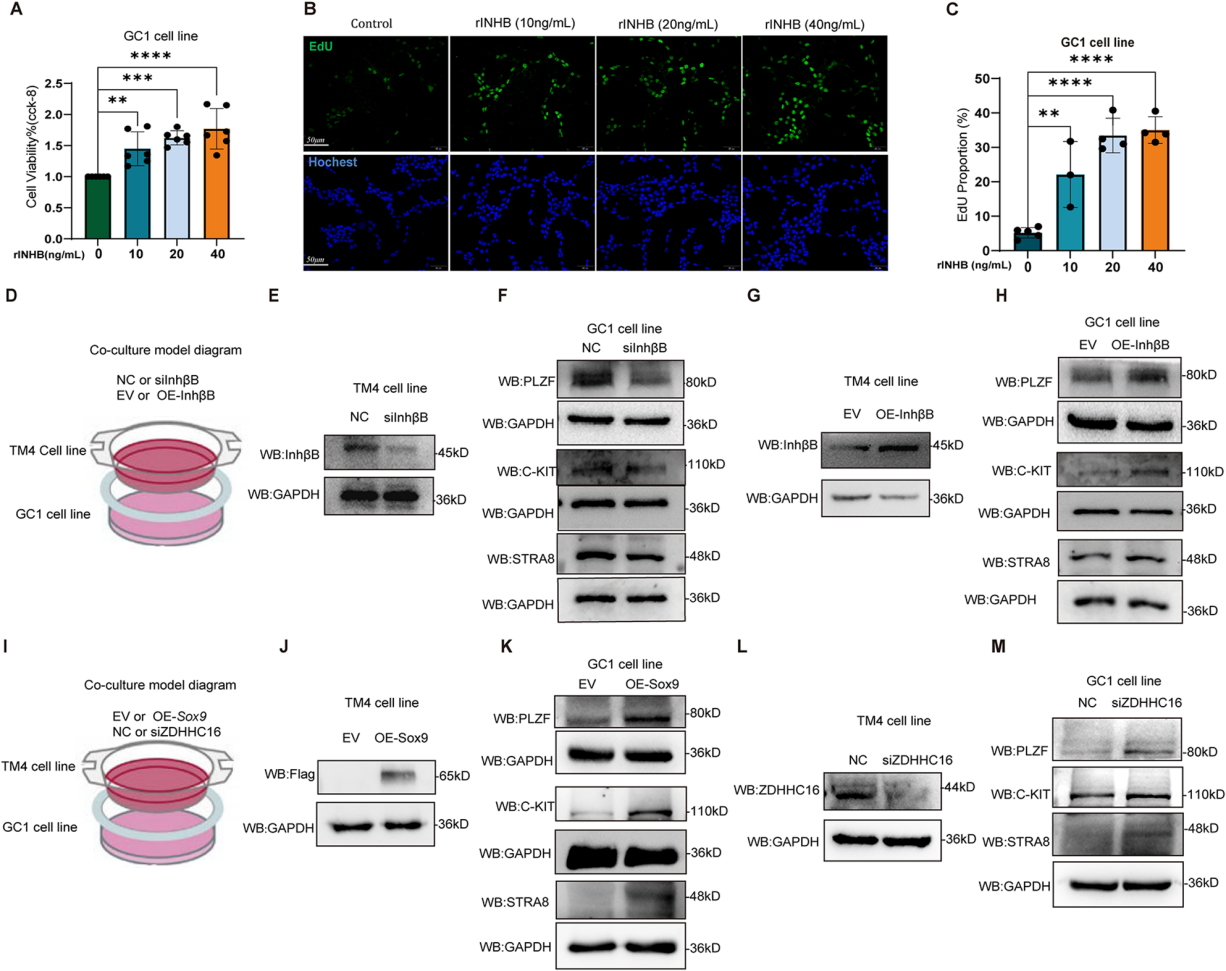
Disrupted INHB synthesis induced by Sox9 hyper-palmitoylation affects spermatogonial proliferation and differentiation. **A** The viability of GC1 was assessed by CCK-8 assay. GC1 cells were treated with recombinant INHB (0, 10, 20, 40 ng/ml) for 48 h (*n* = 6). **B** After GC1 cells were treated with recombinant INHB (0, 10, 20, 40 ng/ml) for 48 h, the EdU incorporation assay was used to assess GC-1 cell proliferation (scale bar, 50 μm) (*n* = 3). **C** Quantification of the number of EdU-labeled cells in each group. Comparison with control: ***p < 0.001. **D** Schematic diagram of TM4-GC1 co-culture system; **E** and **F** The knockdown effects of InhβB in TM4 cells were verified and the expression levels of PLZF, C-KIT and STRA8 were detected by Western blot, after GC1 cells and TM4 cells were co-cultured for 48 h. **G** and **H** The overexpressing effects of InhβB in TM4 cells were verified and the expression levels of PLZF, C-KIT and STRA8 were detected by Western blot, after GC1 cells and TM4 cells were co-cultured for 48 h. **I** Schematic diagram of TM4-GC1 co-culture system. **J** and **K** The overexpressing effects of Sox9*-*Flag (tag) in TM4 cells were verified and the expression levels of PLZF, C-KIT and STRA8 were detected by Western blot, after GC1 cells and TM4 cells were co-cultured for 48 h. **L** and **M** The knockdown effects of ZDHHC16 in TM4 cells were verified and the expression levels of PLZF, C-KIT and STRA8 were detected by Western blot, after GC1 cells and TM4 cells were co-cultured for 48 h

The role of INHB in regulating spermatogonia within the testicular spermatogenic microenvironment remains unclear. To address this, we evaluated the effect of INHB on the proliferation of GC1 cells, a spermatogonial cell line. We treated GC1 cells with various concentrations of recombinant INHB (rINHB; 10, 20, and 40 ng/mL) for 48 h and assessed cell viability and proliferation using CCK-8 and EdU assays respectively. The results showed that GC1 cell viability and proliferation were increased by rINHB in a dose-dependent manner, with an optimal concentration of 40 ng/mL (Fig. 6A–C). This suggests that Sertoli cell-derived INHB may regulate spermatogonial proliferation.

To elucidate the paracrine regulation of spermatogonial dynamics by Sertoli cells through INHB, we established a TM4 (Sertoli cell)-GC1 (spermatogonia) co-culture system, as shown in Fig. 6D. We performed InhβB knockdown or overexpression in TM4 cells using a Flag-tagged InhβB overexpressing plasmid or siRNA specifically targeting *InhβB* gene, respectively (Fig. 6E, G). After transfection, the TM4 cells were co-cultured with GC1 cells for 48 h. Results showed that InhβB knockdown in TM4 cells significantly decreased the expression of both undifferentiated (PLZF) and differentiated (C-KIT, STRA8) spermatogonial markers in GC1 cells (Fig. 3F). In contrast, InhβB overexpression in TM4 cells increased PLZF, C-KIT, and STRA8 expression in GC1 cells (Fig. 6H). These findings indicate that Sertoli cells regulate spermatogonial proliferation and differentiation by secreting INHB.

Furthermore, to elucidate the paracrine regulation of spermatogonial dynamics by Sertoli cells through Sox9 and the ZDHHC16, we used the TM4-GC1 co-culture system again (Fig. 6I). We transfected TM4 cells with a Flag-tagged Sox9 overexpressing plasmid or siRNA specifically targeting *Zdhhc16 gene* (Fig. 6J, L), and then co-cultured the TM4 cells with GC1 cells for 48 h. Results showed that Sox9 overexpression, as well as ZDHHC16 knockdown in TM4 cells, significantly increased the expression of both undifferentiated (PLZF) and differentiated (C-KIT, STRA8) spermatogonial markers in GC1 cells (Fig. 6K, M). These findings indicate that Sertoli cells regulate spermatogonial proliferation and differentiation through the ZDHHC16-Sox9-INHB signaling axis.

## Discussion

**Fig. 7:**
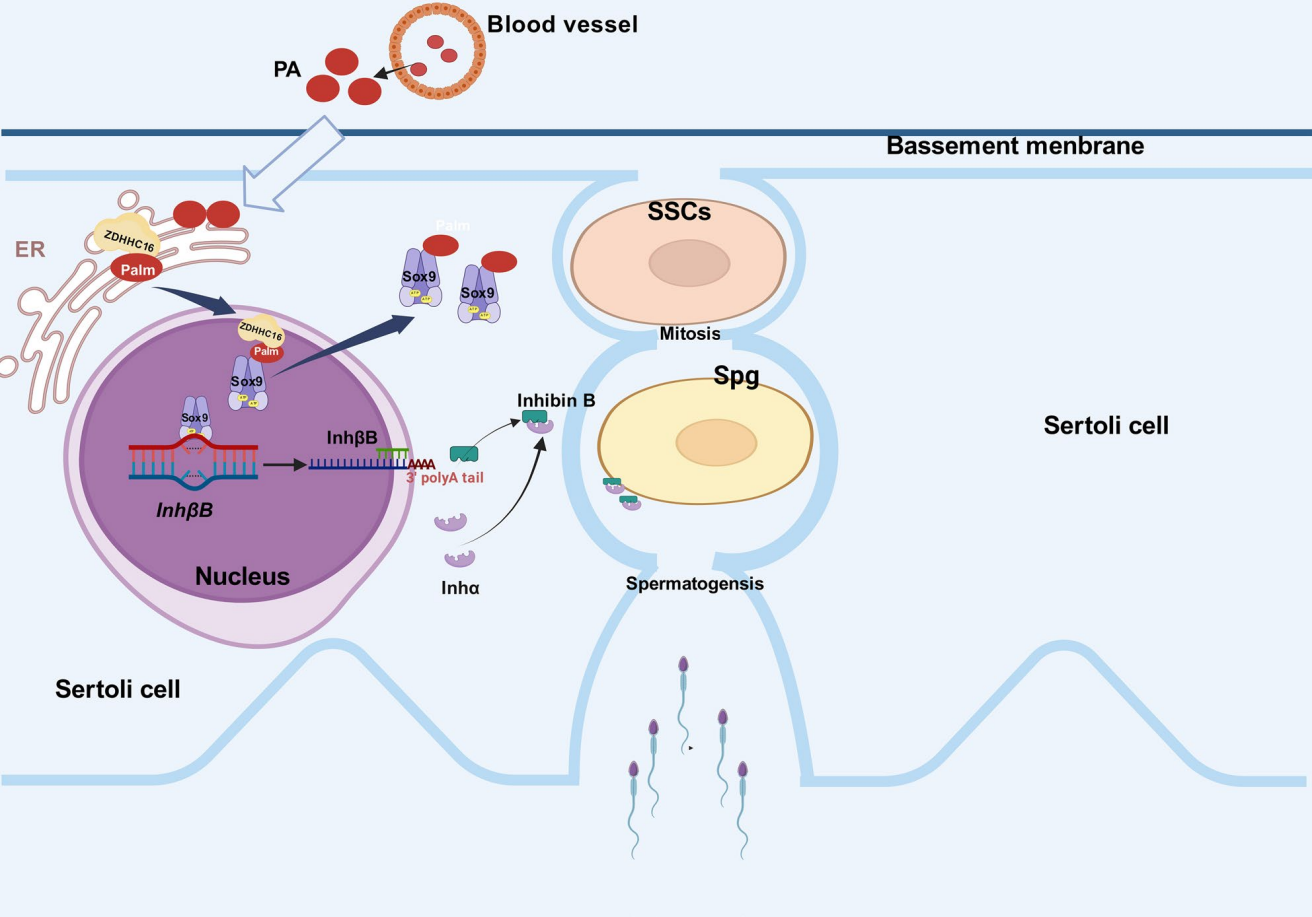
The graph shows how PA affects INHB production by modulating palmitoylation modification of Sox9, which further affects spermatogenesis

In this study, we demonstrated that excess PA reduces INHB synthesis in Sertoli cells by inhibitin*g* the transcription of its critical subunit *InhβB* gene, and PA-induced hyper-palmitoylation of Sox9, which is mediated by ZDHHC16, is involved in this process. Furthermore, the reduction of INHB is responsible for the suppression of spermatogonia proliferation and differentiation in a paracrine manner in the spermatogenesis microenvironment (Fig. 7).

PA is a main component of dietary saturated fatty acid, and is also the most frequent saturated fatty acid in organisms, which has been reported to be a risk factor involved in metabolic syndrome, cardiovascular disease, cancer, as well as reproductive disorder including dyszoospermia [17]. Our previous published study indicated that PA-induced aberrant protein palmitoylation affects Sertoli cell barrier function [17]. In this study, we focused on INHB, a critical hormone secreted by Sertoli cells and a marker of spermatogenesis. In accordance, we found that high PA leads to decreased INHB levels in serum (Fig. 2B). Interestingly, the palmitoylation inhibitor 2BP was found to rescue INHB levels in the testes, but this rescuing effect was not significant in the serum (Fig. 2C), which led us to study the effect of palmitoylation on INHB production in the testis microenvironment, where INHB is synthesized by Sertoli cells. Moreover, in Sertoli cells, PA treatment inhibited the expression of the key subunit of INHB, InhβB mRNA, and therefore down-regulated the production and secretion of INHB, while 2BP exhibited a rescuing effect. These results indicate that PA-induced protein hyper-palmitoylation in Sertoli cells may suppress the synthesis of INHB by inhibiting the transcription of *InhβB* gene.

According to these results, it is likely that PA-induced palmitoylation may affect some transcriptional factors regulating InhβB mRNA expression. Therefore, we identified Sox9. Sox9 is a highly expressed transcriptional factor in testis, which is considered as a Sertoli cell marker. It played an important role in sex determination and Sertoli cell differentiation and maintenance, as well as regulation of spermatogenesis by modulating adult Sertoli cell function [37]. Previously, Sox9 has been proved to promote the transcription of *InhβB* gene by directly binding to its enhancer [38]. In our study, the impacts of PA and 2BP on the expression of InhβB mRNA have been demonstrated to be mediated by Sox9 (Fig. 4). Furthermore, palmitoylation of Sox9 is verified to be regulated by PA and 2BP, and such a post-translational modification is proved to play a vital role in the regulation of Sox9 nuclear localization, which may lead to the alteration of the expression of InhβB mRNA and INHB production (Fig. 3). Whether Sox9 palmitoylation directly modulates *InhβB* gene transcription remains to be fully elucidated. Future work involving site-specific *Sox9* mutants combined with ChIP-qPCR would provide crucial insights into this direct regulatory relationship.

Palmitoylation is mediated by the ZDHHCs [39]. In this study, we identified ZDHHC16 as the palmitoylationansferase mediating PA-induced hyper-palmitoyaltion of Sox9. ZDHHC16 is a palmitoyltransferase reported to play roles in DNA damage response, cell proliferation, ferroptosis and so on [40–42]. Our research revealed that ZDHHC16 affected the nuclear translocation of Sox9 and the synthesis of INHB. While our research findings suggest that ZDHHC16 plays a role in regulating Sox9, more evidences of a direct physical interaction between them is still needed. To substantiate this proposed mechanism, further exploration utilizing co-immunoprecipitation or proximity-dependent ligation assays will be indispensable. These results proposed a novel role of ZDHHC16 in hormone production and subsequent regulation of spermatogenesis.

INHB is commonly considered as a hormone regulating FSH secretion, which controls spermatogenesis. In recent years, the local function of inhibins/activins and the INHB subunit InhβB in microenvironments has also been concerned. For example, knockdown of InhβB was found to increase apoptosis and suppress steroidogenesis in granulosa cells [43]. In tumors, InhβB is becoming a novel potential tumor biomarker, due to its role in cancer cell growth, invasion, metastasis, apoptosis and so on, although the effects were mainly owing to its assembly as homodimer (Activin B) but not heterodimer (INHB). Furthermore, the hormones belonging to the same family of INHB, such as activins and inhibin A, has been reported to regulate Leydig cell function in the testes, especially testosterone biosynthesis [44]. Although the levels of INHB in testis have been found to correlate with spermatogenic function [45], whether INHB can directly affect germ cells has never been confirmed. Here, we demonstrated that Sertoli cell-secreted INHB modulates the proliferation and differentiation of spermatogonia by a paracrine manner (Fig. 6). As the production of INHB can be regulated by protein palmitoylation in Sertoli cells, it can be speculated that in the spermatogenesis microenvironment PA may suppress spermatogonial proliferation and differentiation by inhibiting INHB production through promoting palmitoylation. These results appended an important piece of the puzzle about the role of INHB in the local testis, and prompted a critical role of palmitoylation on Sertoli cell paracrine function.

## Conclusion

In this study, we validated the mechanistic axis that PA influences the transcription of *InhβB* gene and subsequent INHB synthesis. It is plausible that this outcome is driven by inducing Sox9 hyper-palmitoylation. Meanwhile, it prompted that INHB contributes to the enhancement of the proliferation and differentiation of spermatogonia through a paracrine manner in the testis microenvironment. Our results propose the involvement of aberrant palmitoylation in the secretion dysfunction of Sertoli cells, and prompt a novel function of INHB in the spermatogenesis microenvironment, which provides new insights into the pathogenesis of dyszoospermia.

## Supplementary Information

Below is the link to the electronic supplementary material.

Supplementary Material 1

Supplementary Material 2

## Data Availability

No datasets were generated or analysed during the current study.

## Abbreviations

PA: Palmitic acid
2BP: 2-bromopalmitate
NEM: N-ethylmaleimide
HAM: Hydroxylamine
TG: Triglyceride
TC: Cholesterol
FSH: Follicle-stimulating hormone
LH: Luteinizing hormone
HPG: Hypothalamic-Pituitary-Gonadal
FBS: Fetal bovine serum
SFA: Saturated fatty acid
siRNA: Short interfering RNA
T: Testosterone
INHB: Inhibin B
4%PFA: 4% Paraformaldehyde
PTM: Protein post-translational modification
NLS: Nuclear localization signals
zDHHCs: zinc finger DHHC-type palmitoyltransferases
Sox9: SRY-box transcription factor
ABE: Acyl biotin exchange
